# Ring finger 220 promotes the stemness and progression of colon cancer cells via Ubiquitin specific peptidase 22-BMI1 axis

**DOI:** 10.1080/21655979.2021.2003664

**Published:** 2021-12-07

**Authors:** Jianwen Yan, Min Tan, Lin Yu, Xichao Jin, Yangcheng Li

**Affiliations:** a Department of Surgery 1, Guilin Tcm Hospital of China, Guilin City, Guangxi Zhuang Autonomous Region, China; b Department of General Surgery, Nantong Tumor Hospital, Nantong City, Jiangsu Province, China

**Keywords:** RNF220, USP22, BMI1, stemness, progression, colon cancer

## Abstract

Colorectal cancer (CRC) is ranked as the third most common malignancy worldwide. Therefore, it is urgent to screen novel and effective molecular drug targets for colorectal cancer therapeutics. In this study, the specific role and related mechanism underlying Ring finger (RNF) 220 in colon cancer were investigated. Firstly, RT-PCR assay was used to compare differences between expression levels of RNF220 in colorectal tumor and normal tissues. Western blot and RT-PCR assays were applied to examine the protein levels of RNF220 in normal colonic mucosa and colorectal cancer cells. We found that RNF220 was upregulated in colorectal cancer in patients and cell models. RNF220 promoted the proliferation and migration, invasion of colorectal cancer cells through BrdU incorporation, clone formation, transwell and wound healing assays. Spheroid formation and western blot assays illustrated that RNF220 promoted the stemness of colorectal cancer cells. Moreover, we found that RNF220 regulated BMI1 expression through USP22 by western blot. Finally, we discovered that RNF220 facilitated tumor growth *in vivo* through establishment of subcutaneous xenograft tumor mice model. In conclusion, RNF220 promoted the stemness and progression of colon cancer cells via the USP22-BMI1 axis.

## Introduction

Colorectal cancer (CRC) is the third most common malignancy worldwide, with approximately 1 million new cases diagnosed and 500,000 deaths each year. Current clinical therapeutic strategies mainly include surgery, chemotherapy and targeted therapy, however, these treatments are prone to recurrence, exhibiting relatively low 5-year survival rate. The main cause of death in colorectal cancer patients is distant invasion and metastasis [1,2]. Recently, ‘cancer stem cell hypothesis’ has been proposed: a few cancer cells can behave like stem cells, with potential of self-renewal, unlimited proliferation and multidirectional differentiation, which can initiate tumor growth, therefore, they were named as ‘cancer stem cells’ (CSC). CSCs are closely related to tumor metastasis, drug resistance and recurrence [3]. Previous study found that microRNA-301b-3p facilitated cell proliferation and migration in colorectal cancer by targeting HOXB1 [4], while in our study, we identified a novel molecule named RNF220, which was aberrantly expressed in colon cancer.

Ring finger (RNF) protein is characterized by its N-terminal Cys3-His-Cys4 RING domain, which binds to ubiquitin and then transfers it to protein substrates. RNF proteins have been validated to be involved with many human diseases. RNF220 is a member of RING finger domain ubiquitin E3 ligases, and RNF220 knockdown inhibited medulloblastoma progression [5]. RNF220 is involved in the activation of Wnt pathway in colon cancer cells and promotes the deubiquitination of β-catenin [6,7]. RNF220 overexpression contributes to the proliferation of leukemia cells by stabilizing CyclinD1 by increasing USP22 expression [8].

Ubiquitin specific peptidase 22 (USP22) is a deubiquitination enzyme (DUB), which is involved in aggressive growth, metastasis, and CSC. USP22 overexpression may lead to death in CRC patients, suggesting USP22 plays an important role in distant metastasis [9]. Interestingly, USP22 has been reported to promote tumor cell stemness and development by stabilizing BMI1 [10].

BMI1 is a key regulatory component of the multi-comb inhibitory complex 1, which is involved in the self-renewal of CSC and maintains CSC stemness. BMI1 overexpression is closely associated with tumor genesis, metastasis, and invasion in various tumor types. BMI1 inhibitors suppress the invasion proliferation as well as migration of colorectal cancer cells, which also exhibit similar effects on other types of tumors [11].

TCGA shows high expression of RNF220 in colon cancer, but the specific role and related mechanism of RNF220 in colon cancer pathogenesis remain unclear. In this study, we hypothesized RNF220 promoted the stemness and progression of colon cancer cells via the USP22-BMI1 axis, with the aim to investigate the specific role and related mechanism of RNF220 in colon cancer.

## Materials and methods

### Tissue sample

A total of 65 colorectal cancer patient cases of colon tissues and adjacent tissue samples were randomly collected at the Nantong Tumor Hospital. Informed written consents were obtained from all patients for samples collection. Protocols were approved by the Ethics Committee of the Nantong Tumor Hospital. and conducted according to the principles expressed in the Declaration of Helsinki [12].

### Cell culture

The human normal colonic mucosa NCM460 cells and CRC cells SW480, HCT116, SW620, SW837 were purchased from the Chinese Academy of Sciences (Shanghai, China). Cells were cultured in medium (DMEM; Gibco, Carlsbad, CA, USA) supplemented with 10% fetal bovine serum (Gibco), and 1% penicillin/streptomycin (Gibco) in a humidified 5% CO_2_ incubator at 37°C [13].

### qPCR

Total RNAs were extracted by Trizol (Invitrogen, CA, USA) in line with the protocol [13]. Relative mRNA level of RNF220 was analyzed using the 2^−ΔΔCt^ method [14,15]. Primer sequences are listed in Table 1.

**Table 1.** Primers for RNF220 and reference genes

Gene	Primer	Sequence (5ʹ→3ʹ)
RNF220	Forward	TGTGGGCAGAAGCGGATAC
	Reverse	TGTCATCTCCATCCACATCCAG
U6	Forward	TCCTCCACGACAACCAAAACC
	Reverse	TCTTTTCCCAAAATCCCAGACTC
GAPDH	Forward	CTGGGCTACACTGAGCACC
	Reverse	AAGTGGTCGTTGAGGGCAATG

### Western blot

Total proteins were extracted from using RIPA buffer (Beyotime, Shanghai, China). BCA protein assay kit (CoWin Biotechnology) was used to examine protein concentrations. Protein was separated by SDS-PAGE, and transferred onto PVDF membrane. Membranes were incubated with following primary antibodies RNF220 (ab69357, 1:700; Abcam, Cambridge, MA, USA), SOX2 (ab92494, 1:1500; Abcam, Cambridge, MA, USA), OCT4 (ab17929, 1:1000; Abcam, Cambridge, MA, USA), NANOG (ab109250, 1:5000; Abcam, Cambridge, MA, USA), USP22 (ab195289, 1:2000; Abcam, Cambridge, MA, USA), BMI1 (ab126783, 1:30,000; Abcam, Cambridge, MA, USA), p65 (ab32536, 1:10,000; Abcam, Cambridge, MA, USA), PCNA (ab18197, 1:5000; Abcam, Cambridge, MA, USA), and β-actin (ab8227, 1:3000; Abcam, Cambridge, MA, USA) overnight at 4°C. Then, membranes were incubated with HRP-conjugated goat anti-rabbit immunoglobulin G secondary antibody (ab205718, 1:3000; Abcam). β-actin was used as an internal control to normalize the analyzed samples [16].

### Cell transfection

Sh-RNF220 and pcDNA-RNF220, pcDNA-USP22 vector (Genecreate, China) were used to knock down or overexpress RNF220 and USP22 levels. Cells were seeded in 6-well plates for 24 hrs and transfected by Lipofectamine 3000 following manufacture’s protocol (Thermo Fisher Scientific, Grand Island, USA) [17].

### BrdU incorporation assay

Transfected SW480 cells were inoculated on the cover slides placed on 6-well plates (1*10^6^ cells/well). 20 hrs-post-seeding, bromodeoxyuridine (BrdU) were added. Cells were then incubated overnight at 4°C with mouse monoclonal antibodies against BrdU (1:200, Cell Signaling Technology, Beverly. MA, USA).After three times wash with 1xPBS, cells were labeled with an anti-mouse secondary antibody conjugated with Alexa Fluor 594 (1:200, Thermo Fisher Scientific, Waltham, Ma, USA), and the nuclei were labeled with 4.6-didipriinyl-2-phenyl indoles (DAPI, Themt Fisher Scientific, Waltham, MA, USA) [18].

### Cloning formation assay

Crystal violet staining method was used to assess cell proliferation ability [19]. Briefly, 5*10^3^ SW480 cells were seeded into 6-well plates and treated in different conditions. 3 weeks post-seeding, supernatant was removed and then cells were fixed with 4% formaldehyde for 15 minutes. Finally, cells were stained with 0.25% crystal violet solution for 25 minutes. After drying the culture plate, we counted the cells number.

### Wound healing assay

Briefly, 1*10^6^ SW480 cells were treated with different conditions [19]. 18hrs post-seeding, we used a pipette tip and a ruler to scratch the horizontal line on the surface of the plate. Finally, we rinsed the plate 3 times by 1xPBS and cultured the cells in 5% CO_2_ incubator. Photos were taken 24 hrs post-incubation.

### Spheroid formation assay

1*10^3^ SW480 cells were cultured in DMEM/F12 medium (Invitrogen, Shanghai, China) contained with 4 mg/mL B27 (1:50, GIBCO, Shanghai, China), insulin (Sigma, Shanghai, China) 20 ng/mL basic FGF (Sigma, Shanghai, China) and 20 ng/mL EGF (Sigma, Shanghai, China). Then, we collected the serial passages of primary spheres, which were dissociated in trypsin, resuspended in DMEM/F12 medium with the above supplements, and plated to generate secondary spheroids. Number of spheres was counted by microscope [20].

### Xenograft experiments

Five-week-old female nude mice (n = 5) were purchased from Shanghai Experimental Animal Center (Shanghai, China), and then were subcutaneously injected with equal numbers of sh-Ctrl and shRNA-RNF220 transfected SW480 cells (5*10^6^). Tumor volume was periodically blindly measured by caliper every 5 days until mice were sacrificed. Mice were executed 30 days post-injection. Then the tumor tissues were obtained [20]. All animal experiments were approved by the Ethics Committee of Guilin TCM Hospital of China. For the use of animals and conducted in accordance with the National Institutes of Health Laboratory Animal Care and Use Guidelines.

### Statistical analysis

All data are exhibited of the mean ± standard deviation (SD) from three independent experiments [20]. Student’s t-test was applied to compute the comparisons between two groups. GraphPad Prism 5 (GraphPad Software, Inc., San Diego, CA, USA) was applied for analysis. A difference P value of <0.05 was considered statistically significant.

## Results

In this study, we hypothesized RNF220 promoted the stemness and progression of colon cancer cells via the USP22-BMI1 axis, with the aim to investigate specific role and related mechanism of RNF220 in colon cancer. We found that RNF220 was highly expressed in colorectal cancer tissues and cells, which facilitated the proliferation, migration and invasion of colorectal cancer cells, and thus promoting the stemness of colorectal cancer cells. RNF220 regulated BMI1 expression through USP22, promoting tumor growth *in vivo*.

### RNF220 was highly expressed in colorectal cancer tissues and cells

**Figure 1.**
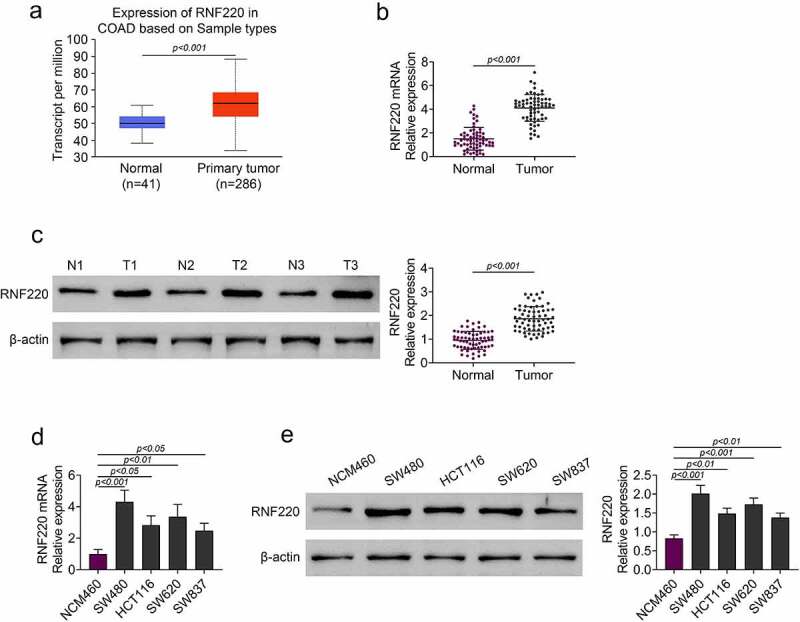
RNF220 was highly expressed in colorectal cancer tissues and cells. (a) TCGA database showed the expression level of RNF220 in primary colorectal tumor (n = 286) and normal tissues (n = 41), p < 0.001. (b-c) RT-PCR and western blot analysis showed the RNF220 mRNA and protein level in colorectal tumor and para-carcinoma tissue from 65 patients, p < 0.001, β-actin was normalized as an internal control. (d-e) RT-PCR and western blot analysis showed the RNF220 protein level in normal colonic mucosa NCM460 cells and colorectal cancer cells SW480, HCT116, SW620 and SW837, p < 0.05, p < 0.01, p < 0.001. All representative data are from three independent experiments. β-actin was normalized as an internal control

According to TCGA database, RNF220 expression level was elevated in colorectal tumor (n = 286) compared with that in normal tissue (n = 41) (Figure 1a). Next, we discovered the mRNA and protein levels of RNF220 were upregulated in colorectal tumor compared with those in para-carcinoma tissue from 65 patients (Figure 1b and c). We also examined the RNF220 expression level in normal colonic mucosa NCM460 cells and colorectal cancer cells SW480, HCT116, SW620 and SW837. RT-PCR and western blot assays demonstrated RNF220 expression was notably increased in SW480, HCT116, SW620 and SW837 cells compared to that in NCM460 cells (Figure 1d and e). Taken together, these results revealed that RNF220 was expressed highly in colorectal cancer.

### RNF220 promoted the proliferation, migration and invasion of colorectal cancer cells

**Figure 2.**
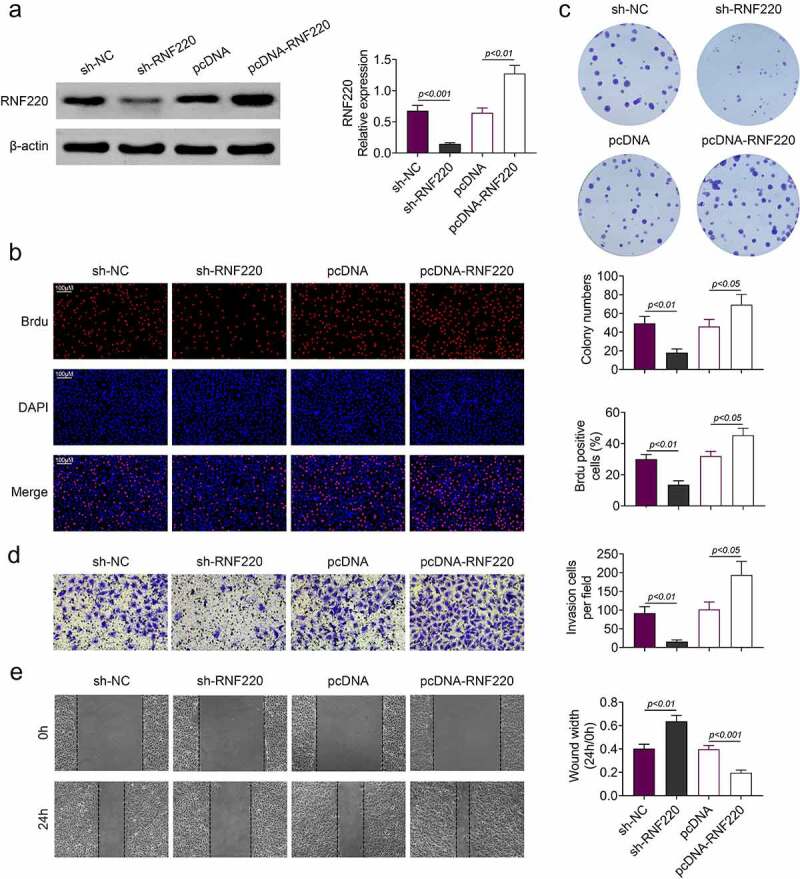
RNF220 promoted the proliferation, migration and invasion of colorectal cancer cells. (a) Western blot assay detected the transfection efficiency, p < 0.01, p < 0.001. β-actin was normalized as an internal control. (b-c) Brdu and colon formation assays dectected the SW480 cells proliferasion abilities in sh-NC, sh-RNF220, pcDNA and pcDNA-RNF220 groups. p < 0.05, p < 0.01. (d-e) Transwell and wound healing assays examined the SW480 cells invasion and migration in sh-NC, sh-RNF220, pcDNA and pcDNA-RNF220 groups. p < 0.05, p < 0.01, p < 0.001. All representative data are from three independent experiments

Subsequently, *in vitro* assays were employed to assess the functions of RNF220 in colorectal cancer. In western blot assay, expression level of RNF220 was significantly decreased in sh-RNF220 group compared to that in sh-NC group. In contrast, RNF220 level was increased in pcDNA-RNF220 group compared to that in pcDNA group (Figure 2a). Next. we discovered that the proliferation ability of SW480 cells was inhibited in sh-RNF220 group, to the contrary, the proliferation ability of SW480 cells was strengthened in pcDNA-RNF220 group, according to Brdu and colon formation assyas (Figure 2b and c). Transwell and wound healing assays results suggested SW480 cells invasion and migration were decreased upon sh-RNF220 transfection, while the invasive and migrative abilities of SW480 cells were elevated upon pcDNA-RNF220 transfection (Figure 2d and e). Together, these results demonstrated that RNF220 promoted the proliferation, invasion and migration in colorectal cancer cell models.

### RNF220 promotes the stemness of colorectal cancer cells

**Figure 3.**
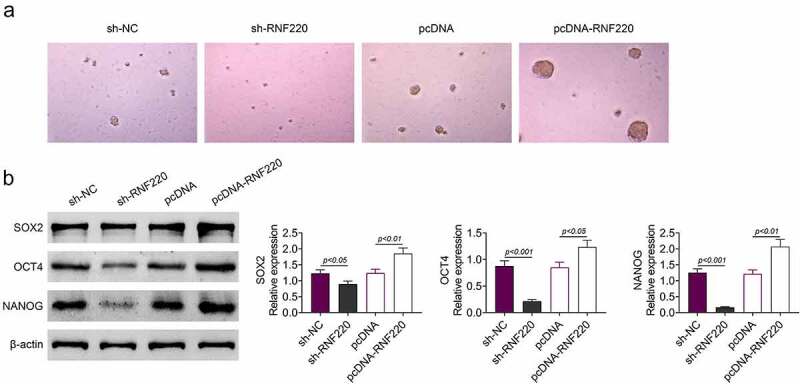
RNF220 promotes the stemness of colorectal cancer cells. (a) Representative images of sphere formation induced by the transfection of sh-NC, sh-RNF220, pcDNA and pcDNA-RNF220 plasmids into SW480 cells. (b) Western blot analysis shows the expression levels of SOX2, OCT4 and NANOG in sh-NC, sh-RNF220, pcDNA and pcDNA-RNF220 groups, p < 0.05, p < 0.01, p < 0.001. All representative data are from three independent experiments. β-actin was normalized as an internal control

To further explore the precise functions of RNF220 in the stemness of colorectal cancer cell, spheroid formation assay was utilized. As shown in Figure 3a, RNF220 knockdown notably suppressed the sphere-propagating capacity, while RNF220 overexpression strengthened the sphere-propagating ability in colorectal cancer cell SW480. Western blot illustrated protein levels of SOX2, OCT4 and NANOG were reduced in sh-RNF220 group compared to those in sh-NC group, these protein markers were increased in pcDNA-RNF220 group compared to those in pcDNA group (Figure 3b). To sum up, RNF220 promoted the stemness of colorectal cancer cells.

### RNF220 regulates BMI1 expression through USP22

**Figure 4.**
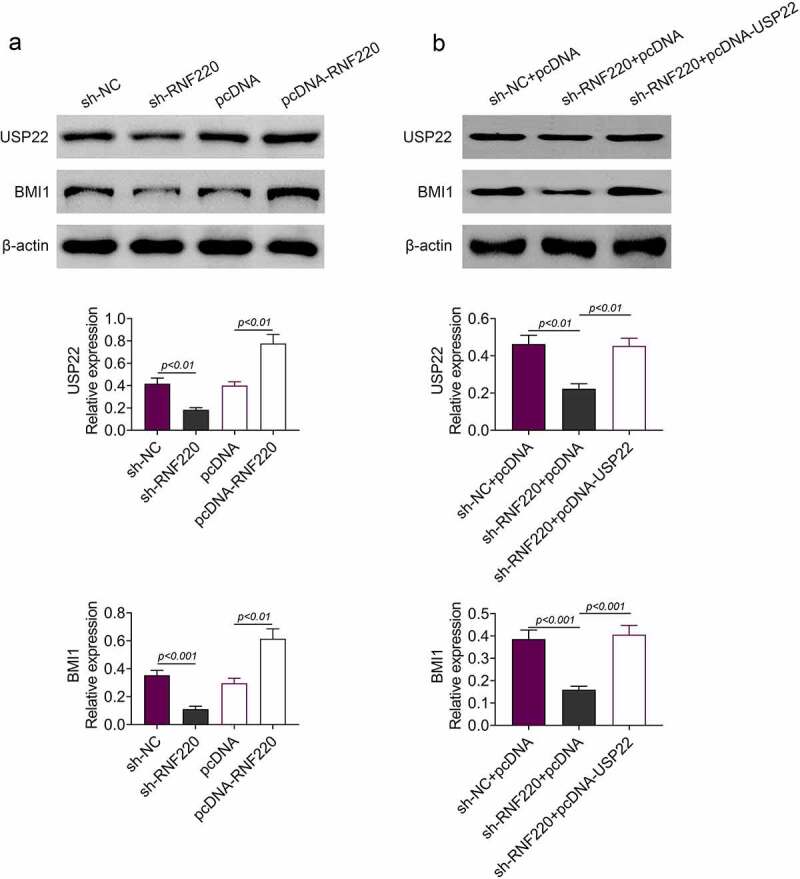
RNF220 regulates BMI1 expression through USP22. (a) Western blot analysis examined the USP22 and BMI1 expression in SW480 cells transfected with sh-NC, sh-RNF220, pcDNA and pcDNA-RNF220 plasmids, p < 0.01, p < 0.001. β-actin was normalized as an internal control. (b) Western blot analysis examined the USP22 and BMI1 expression in SW480 cells transfected with sh-NC+pcDNA, sh- RNF220+ pcDNA and sh-RNF220+ pcDNA-USP22 plasmids, p < 0.01, p < 0.001. All representative data are from three independent experiments. β-actin was normalized as an internal control

According to western blot analysis, RNF220 knockdown inhibited protein expression levels of BMI1 and USP22 in SW480 cells, similarly, RNF220 overexpression boosted their protein levels (Figure 4a). Moreover, USP22 and BMI1 levels were decreased in cells expressing sh-RNF220+ pcDNA compared to those in cells expressing sh-NC+pcDNA. Protein levels of USP22 and BMI1 could be restored upon expression of sh-RNF220+ pcDNA-USP22 (Figure 4b). Collectively, these data demonstrated that RNF220 regulated the expression of BMI1 through USP22.

### *RNF220 promotes tumor growth* in vivo

**Figure 5.**
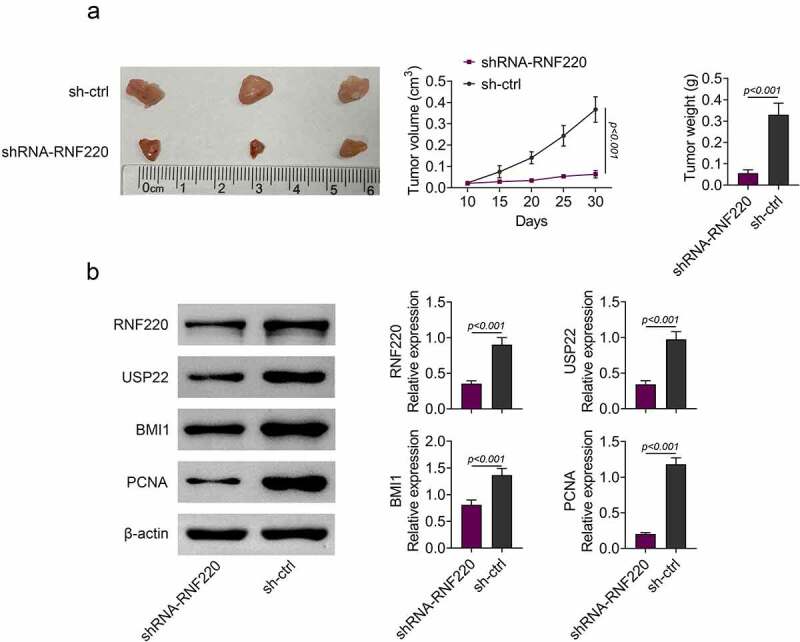
RNF220 promotes tumor growth *in vivo*. (a) Tumors were obtatined from sh-ctrl and shRNA-RNF220 groups of subcutaneous xenograft mice model (n = 5) and tumors volume and weight were measured after injection for 30 days, p < 0.001. (b) The expression of RNF220, USP22, BMI1 and PCNA in shRNA-RNF220 group mice and sh-ctrl group mice were detected by western blot, p < 0.001. All representative data are from three independent experiments. β-actin was normalized as an internal control

To validate functions of RNF220 *in vivo*, we established the subcutaneous xenograft tumor mice model which stably knocked down RNF220 (n = 5). We observed smaller tumor sizes in shRNA-RNF220 group compared to those in sh-Ctrl group. Furthermore, 30 days-post injection, the tumor volume and weight were significantly decreased in shRNA-RNF220 group, suggesting an oncogenic role of RNF220 (Figure 5a). We also discovered that protein levels of RNF220, USP22, BMI1 and PCNA were significantly reduced in shRNA-RNF220 group mice (Figure 5b). Taken together, RNF220 promoted tumor growth *in vivo*.

## Discussion

Even though therapeutic strategies targeting cancer have made great progresses in the past few decades, the precise pathogenic mechanisms of colorectal cancer remain unclear. Recently, several studies report RNF220 regulates progression in various types of cancers. RNF220 mediates medulloblastoma development by epigenetic modulation of Shh signal pathway [5], as well as accelerates the leukemic cells proliferation and declines the Cyclin D1 protein degradation [8]. However, the effects of RNF220 on colorectal cancer fate determination remains blearily. In this study, we discovered that RNF220 level was elevated in colorectal cancer, promoting the migration, invasion and proliferation of colorectal cancer cells through USP22-BMI1 axis. These data suggested that RNF220 might function as a regulatory molecule in the processes of colorectal cancer.

Considered as an oncogenic factor, the deubiquitinase USP22 links to tumor development. USP22 regulates PD-L1 degradation in several human cancer cells [21]. USP22 promoted cancer progression by regulating cell proliferation and DNA repair in prostate cancer [22]. USP22 also promoted gastric cancer progression and metastasis through regulating c-Myc/NAMPT/SIRT1-dependent FOXO1 and YAP signaling [23]. USP22 was also been reported to accelerate the development of cancer cells by targeting the DYRK1A in pancreatic ductal adenocarcinoma [24]. However, the functions of USP22 in colorectal cancer have not been investigated. USP22-dependent HSP90AB1 expression contributed to resistance to HSP90 suppression in colorectal cancer [25]. USP22 controls secreted protein acidic and rich in cysteine expression and inflammation intensity in colorectal cancer [26]. Our study discovered USP22 combined with BMI1, and thus accelerating stemness of colorectal cancer. It is noteworthy that RNF220 regulates BMI1 expression via USP22, therefore, USP22-BMI1 axis was validated in this study to function in colorectal cancer progression.

USP22-BMI1 axis are reported to be involved in the stemness of many types of cancer cells. Hypoxia-induced USP22-BMI1 axis accelerates the malignancy and stemness of glioma stem cells by regulating the HIF-1α [16]. USP22 maintains stemness of CSC and promotes gastric cancer development through stabilizing the BMI1 protein [10]. Co-expression of USP22 and BMI1 could accelerate tumor development, stemness and predict therapy failure in gastric carcinoma [27]. In this study, we found that RNF220 promoted the stemness and progression of colon cancer cells via the USP22-BMI1 axis. Furthermore, our *in vivo* model suggested that RNF220 promoted tumor growth by regulating the USP22-BMI1 axis.

Taken together, we discovered RNF220 expressed highly in colorectal cancer. RNF220 promoted the proliferation, migration and invasion of colorectal cancer cells. Meanwhile, RNF220 contributed to the stemness of colorectal cancer cells. Interestingly, RNF220 regulated BMI1 expression through USP22. We validated the role of RNF220 in vivo and in vitro, demonstrating RNF220 promoted colorectal cancer growth. All these results illustrated the pro-tumorigenic effects of RNF220/USP22/BMI1 signal pathway on proliferation and stemness of colorectal cancer, providing novel insights into RNF220 as clinical therapeutic candidates for future drug design and development targeting colorectal cancer.

## Conclusion

In this study, we observed RNF220 was highly expressed in colorectal cancer cells and tissues. RNF220 promoted the invasion, migration and proliferation of colorectal cancer cells, by contributing to stemness of cancer cells. Moreover, RNF220 regulated BMI1 expression through USP22. Finally, we found that RNF220 facilitated tumor development *in vivo*. Taken together, we discovered that RNF220 functioned as an oncogenic factor by promoting the stemness the progression of colon cancer via the USP22-BMI1 axis, shedding lights on RNF220 as a potential therapeutic targets for colon cancer treatment from bench to clinic.

## Data Availability

All data generated or analyzed during this study are included in this published article.

## Highlight

- RNF220 was highly expressed in colorectal cancer tissues and cell models
- RNF220 promoted the proliferation, migration and invasion of colorectal cancer cells
- RNF220 promoted the stemness of colorectal cancer cells
- RNF220 regulated BMI1 expression through USP22
- RNF220 promoted tumor growth in vivo
